# Serum ferritin as a prognostic biomarker in CAR-T therapy for multiple myeloma: A meta-analysis

**DOI:** 10.17305/bb.2025.12129

**Published:** 2025-03-28

**Authors:** Jing Cheng, Yuan Song

**Affiliations:** 1 Department of Hematology, The Second Affiliated Hospital, Jiangxi Medical College, Nanchang University, Donghu District, Nanchang City, Jiangxi Province, China

**Keywords:** Multiple myeloma, chimeric antigen receptor-modified T cells, CAR-T, ferritin, survival, progression

## Abstract

Serum ferritin, a marker of systemic inflammation and iron metabolism, has been implicated in the outcomes of patients with relapsed/refractory multiple myeloma (R/R MM). However, its prognostic significance in R/R MM patients undergoing chimeric antigen receptor-modified T-cell (CAR-T) therapy remains unclear. This meta-analysis aimed to evaluate the association between pre-infusion serum ferritin levels and survival outcomes in R/R MM patients treated with CAR-T therapy. We systematically searched PubMed, Embase, and Web of Science for relevant studies. Studies reporting progression-free survival (PFS) and/or overall survival (OS) based on serum ferritin levels were included. Hazard ratios (HRs) with 95% confidence intervals (CIs) were pooled using a random-effects model. Eight retrospective cohort studies, encompassing 1077 patients, met the inclusion criteria. High pre-infusion serum ferritin levels were significantly associated with worse PFS (HR: 2.15, 95% CI: 1.74–2.66, *P* < 0.001) and OS (HR: 2.86, 95% CI: 2.20–3.72, *P* < 0.001), with mild heterogeneity (*I*^2^ ═ 9% for PFS and 0% for OS). Sensitivity analyses, conducted by excluding one study at a time, confirmed the robustness of these findings. Subgroup analyses showed consistent results across different CAR-T product sources (commercial vs academic), ferritin cutoffs, and follow-up durations (*P* for subgroup differences all >0.05). In conclusion, elevated serum ferritin levels before CAR-T infusion predict poorer survival outcomes in R/R MM patients. These findings highlight the potential prognostic value of ferritin and its role in optimizing patient selection and management strategies in CAR-T therapy.

## Introduction

Relapsed/refractory multiple myeloma (R/R MM) is a challenging hematologic malignancy marked by disease persistence or recurrence despite standard therapies [1, 2]. This aggressive form of myeloma contributes significantly to morbidity and mortality among patients [3]. The prognosis remains poor, especially for those who have failed multiple lines of treatment, with limited therapeutic options available [4]. In recent years, chimeric antigen receptor T-cell (CAR-T) therapy has emerged as a promising approach, offering targeted and durable responses by redirecting a patient’s immune cells to attack malignant plasma cells expressing B-cell maturation antigen (BCMA) [5, 6]. Despite its efficacy, CAR-T therapy outcomes vary widely, highlighting the need for a deeper understanding of prognostic factors to guide patient selection and improve treatment outcomes [7, 8].

Ferritin, a ubiquitous iron-storage protein, plays a dual role as both a key regulator of iron homeostasis and an acute-phase reactant [9]. Elevated serum ferritin levels are frequently associated with systemic inflammation, oxidative stress, and immune dysregulation [10, 11]—factors commonly observed in advanced malignancies and intensive therapies such as CAR-T. In the context of R/R MM, ferritin’s role may extend beyond its biochemical properties, potentially serving as a biomarker for disease activity and treatment outcomes [12]. Previous studies have suggested that elevated ferritin levels may correlate with adverse clinical outcomes, including reduced survival, though evidence specific to patients undergoing CAR-T therapy remains limited [13, 14]. The potential mechanisms linking high serum ferritin to poor prognosis in CAR-T–treated R/R MM patients are multifaceted. Elevated ferritin may reflect a pro-inflammatory milieu that worsens treatment-related toxicities, such as cytokine release syndrome (CRS) [15] and immune effector cell–associated neurotoxicity syndrome (ICANS) [16]—both critical determinants of CAR-T outcomes. Additionally, ferritin’s association with iron overload and oxidative stress may impair immune cell function, diminishing CAR-T cell efficacy and contributing to an immunosuppressive tumor microenvironment [17]. Despite these insights, the prognostic value of serum ferritin in the context of CAR-T therapy for R/R MM remains poorly defined, with most available evidence derived from small, heterogeneous studies [18–25]. To address this knowledge gap, we conducted a meta-analysis to comprehensively evaluate the association between pre-infusion serum ferritin levels and survival outcomes in patients with R/R MM treated with CAR-T therapy.

## Materials and methods

### Study design and data sources

This meta-analysis was conducted in accordance with the Preferred Reporting Items for Systematic Reviews and Meta-Analyses (PRISMA) guidelines [26, 27] and the Cochrane Handbook for Systematic Reviews of Interventions [28]. The protocol was registered in the International Prospective Register of Systematic Reviews (PROSPERO) under the identifier CRD42025636605. We systematically searched PubMed, EMBASE, and Web of Science from database inception to January 3, 2025. The search strategy included terms related to “ferritin,” “CAR-T therapy,” and “relapsed/refractory multiple myeloma,” using the following combination: (“ferritin” OR “ferritins”) AND (“myeloma” OR “multiple myeloma”) AND (“Chimeric Antigen Receptor” OR “Chimeric Antigen Receptors” OR “Chimeric T Cell Receptors” OR “Chimeric T-Cell Receptors” OR “Chimeric Antigen Receptor T Cell” OR “CAR-T” OR “Artificial T Cell Receptors” OR “Artificial T-Cell Receptors” OR “chimeric immunoreceptors” OR “Axicabtagene ciloleucel” OR “Axi-cel” OR “KTE-C19” OR “KTEC19” OR “CTL-019” OR “CTL019” OR “Yescarta” OR “Lisocabtagene” OR “maraleucel” OR “Liso-cel” OR “JCAR-017” OR “JCAR017” OR “Breyanzi” OR “Brexucabtagene” OR “autoleucel” OR “Brexu-cel” OR “KTE-X19” OR “KTEX19” OR “Tecartus” OR “Tisagenlecleucel” OR “Tisa-cel” OR “Kymriah” OR “ART-19” OR “CART19” OR “Axicabtagene” OR “ciloleucel” OR “Idecabtagene” OR “vicleucel” OR “Ciltacabtegene” OR “autoleucel”). Reference lists of relevant original and review articles were also screened to identify additional eligible studies. Only studies published in English were included. Detailed search strategies for each database are provided in the Supplemental data.

### Inclusion and exclusion criteria

The inclusion criteria for potential studies were defined according to the PICOS framework:

P (patients): Adults (≥18 years old) diagnosed with R/R MM who were treated with CAR-T.

I (exposure): A high serum ferritin level before the infusion of CAR-T was considered as exposure. The cutoffs for defining a high level of serum ferritin were consistent with the values used in the original studies.

C (comparison): Patients with a low level of serum ferritin before the infusion of CAR-T were considered as controls.

O (outcome): Evaluated the median progression-free survival (PFS) and/or overall survival (OS) following CAR-T therapy by comparing R/R MM patients with high vs low serum level of ferritin and reported the data of the hazard ratio (HR) and 95% confidence interval (CI) for these outcomes. In general, PFS was defined as the time from CAR-T infusion to relapse, disease progression, all-cause mortality, or the last follow-up, while OS was defined as the time from CAR-T infusion to all-cause mortality or the last follow-up.

S (study design): Observational studies with longitudinal follow-up, such as cohort studies, nested case-control studies, and post-hoc analyses of clinical trials.

Review, editorial, meta-analyses, preclinical studies, studies not limited to patients with MM, without serum ferritin level as exposure, or studies did not report the outcomes of interest were excluded. If two or more studies had overlapping populations, the study with the largest sample size was included in the meta-analysis.

### Study quality evaluation and data extraction

The literature search, study identification, quality assessment, and data extraction were conducted independently by two authors. Any disagreements were resolved through discussion and consensus. Study quality was assessed using the Newcastle–Ottawa Scale (NOS) [29], which evaluates selection, control of confounders, and outcome measurement and analysis. NOS scores range from 1 to 9, with nine indicating the highest quality. Selection criteria included: representativeness of the cohort (one point if patients were consecutively or randomly selected), ascertainment of exposure (one point if standard laboratory assays were used), and baseline disease status (one point if the outcome was not present at baseline). Comparability was scored based on adjustments for age and sex (one point) and other key confounders (one point). Outcome assessment included adequate follow-up duration (one point if ≥12 months), completeness of follow-up (one point if ≤20% loss to follow-up), and reliable outcome assessment (one point if based on medical records or registries). Data collected for analysis included study details (author, year, country, and design), patient characteristics (diagnosis, sample size, mean age, and sex), CAR-T products used, timing of serum ferritin measurement, cutoff values used to define high serum ferritin levels, follow-up duration, reported outcomes, and variables adjusted for when analyzing the association between serum ferritin levels and survival outcomes.

### Statistical analysis

The associations between serum ferritin levels and PFS or OS in patients with R/R MM receiving CAR-T therapy were reported as HRs with corresponding 95% CIs. HRs and their standard errors were calculated from 95% CIs or *P* values, followed by logarithmic transformation to stabilize variance and normalize distribution [28]. Heterogeneity was assessed using the Cochrane *Q* test [28], and quantified with the *I*^2^ statistic. An *I*^2^ value of 0% indicates no observed heterogeneity, while values of 25%, 50%, and 75% represent low, moderate, and high heterogeneity, respectively [30]. A random-effects model was employed to pool HRs and 95% CIs, accounting for potential variability among studies [28]. Despite the low statistical heterogeneity observed (*I*^2^ ═ 9% for PFS and 0% for OS), a random-effects model was chosen as a conservative approach due to potential differences in study populations, ferritin cutoffs, and other unmeasured confounders. When heterogeneity is minimal, results from random-effects models are typically similar to those from fixed-effects models, providing robust and generalizable findings [28]. Sensitivity analyses were conducted by sequentially excluding individual studies to assess the robustness of the results. Predefined subgroup analyses were performed based on the source of the CAR-T product (commercial vs academic), ferritin cutoff values, and follow-up durations. For continuous variables, median values were used as subgroup cutoffs. Publication bias was assessed through funnel plots and visual inspection for asymmetry, supplemented by Egger’s regression test [31]. All analyses were conducted using RevMan (version 5.1; Cochrane Collaboration, Oxford, UK) and Stata (version 12.0; StataCorp, College Station, TX, USA).

## Results

### Study inclusion

**Figure 1.**
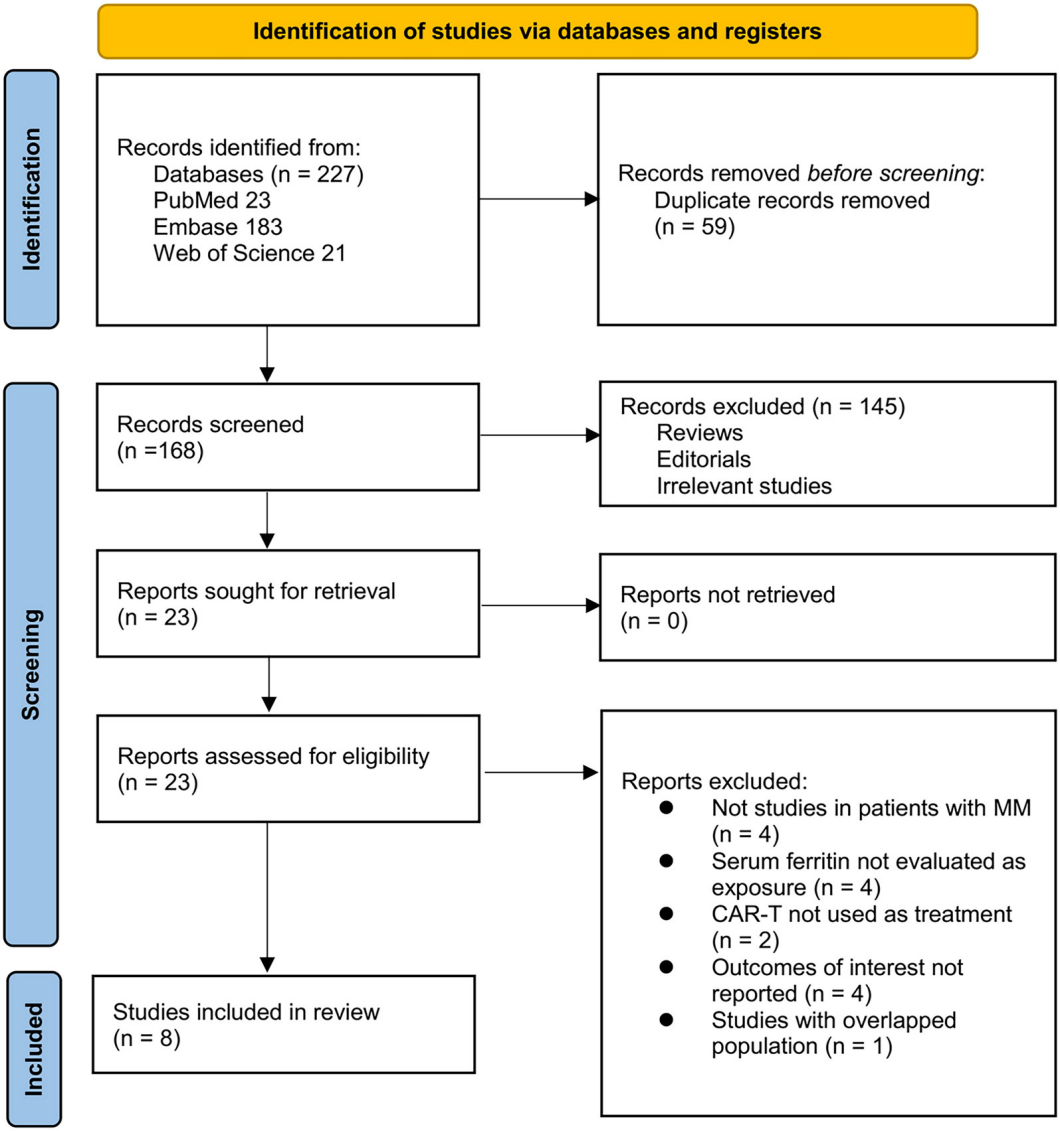
**Flowchart of database search and study inclusion.** CAR-T: Chimeric antigen receptor-modified T-cell; MM: Multiple myeloma.

The study inclusion process is illustrated in Figure 1. A total of 227 potentially relevant records were initially identified through three databases and citation tracking. After removing 59 duplicates, 168 records remained. Title and abstract screening led to the exclusion of 145 articles that did not meet the meta-analysis objectives. The full texts of the remaining 23 articles were then reviewed independently by two authors, resulting in the exclusion of 15 more records for reasons detailed in Figure 1. Ultimately, eight articles were included in the quantitative analysis [18–25].

**Table 1:** Characteristics of the included studies

**Author year**	**Country**	**Study design**	**Diagnosis**	**Sample size**	**Mean age (years)**	**Male (%)**	**CART-T treatment**	**Timing of ferritin measuring**	**Ferritin cutoff determination**	**Ferritin cutoff value (ng/mL)**	**Follow-up duration (months)**	**Outcomes reported**	**Variables adjusted**
Mohty, 2023	USA	RC	R/R MM	133	66	44	Idecabtagene vicleucel or Ciltacabtagene autoleucel	During lymphodepletion	Median	228	9	OS	Age, sex, CRP, CAR-T product, marrow burden, R-ISS, EMD, use of bridging therapy, and ECOG ≥2
Liu, 2023	China	RC	R/R MM	109	57	59	Academic anti-BCMA CAR-T cells alone or combined with anti-CD19 CAR-T cells	Within 3 days before CAR-T cell infusion	Upper quartile	882.3	32	PFS and OS	Age, sex, tumor burden, EMD, and R-ISS stage
Dima, 2024	USA	RC	R/R MM	152	63	54	Idecabtagene vicleucel or Ciltacabtagene autoleucel	Before lymphodepletion and CAR-T infusion	ULN	400	12.5	PFS and OS	Age, sex, active EMD, ECOG PS, cytogenetics, penta-refractory status, prior BCMA therapy, and baseline CRP
Moreno, 2024	USA	RC	R/R MM	40	64	62.5	Idecabtagene vicleucel or Ciltacabtagene autoleucel	Pre-CAR-T infusion	Median	337	13.5	PFS and OS	None
Hashmi, 2024	USA	RC	R/R MM	211	64	60	Idecabtagene vicleucel	During lymphodepletion	ULN	400	9.9	PFS and OS	Age, sex, prior BCMA therapy, EMD, bridging therapy, ethnicity, and t(4;14) at infusion
Gagelmann, 2024	Spain and Israel	RC	R/R MM	60	63	54	Academic CAR-T products targeting BCMA	Before lymphodepletion	ULN	400	10.7	PFS and OS	Age, sex, EMD, high-risk cytogenetics, lenalidomide refractoriness, and MyCARe risk
Davis, 2024	USA	RC	R/R MM	136	62	53	Idecabtagene vicleucel or Ciltacabtagene autoleucel	Before lymphodepletion	ULN	400	7	PFS and OS	Age, sex, frailty, type of CAR-T product, HCT-CI, ECOG PS, EMD, high-risk cytogenetics, and penta-refractory status
Sidana, 2025	USA	RC	R/R MM	236	64	57	Ciltacabtagene autoleucel	Before lymphodepletion	ULN	400	13	PFS and OS	Age, sex, prior BCMA targeted therapy, ECOG PS, EMD, and high-risk cytogenetics

BCMA: B-cell maturation antigen; CAR-T: Chimeric antigen receptor T-cell; CRP: C-reactive protein; EMD: Extramedullary disease; ECOG PS: Eastern Cooperative Oncology Group performance status; HCT-CI: The Hematopoietic Cell Transplantation Comorbidity Index; MyCARe: Myeloma Comorbidity and Age Risk Evaluation; OS: Overall survival; PFS: Progression-free survival; R-ISS: The Revised International Staging System; RC: Retrospective cohort; R/R MM: Relapsed/refractory multiple myeloma; ULN: Upper limit of normal.

### Overview of the study characteristics

Table 1 summarizes the characteristics of the studies included in the meta-analysis. All were retrospective cohort studies conducted between 2023 and 2025 in the United States, China, Spain, and Israel. In total, 1077 patients with R/R MM were included, with mean ages ranging from 57.0 to 64.0 years and the proportion of male patients varying from 44.0% to 62.5%. Commercial BCMA-directed CAR-T cell therapies, including Idecabtagene Vicleucel and Ciltacabtagene Autoleucel, were used in six studies [19–21, 23–25], while academic anti-BCMA CAR-T cells were used in two studies [18, 22]. Serum ferritin levels were measured prior to CAR-T infusion in all included studies. Cutoff values for high serum ferritin varied: two studies [19, 24] used the median, five studies [20–23, 25] used the upper limit of normal (ULN), and one study [18] used the upper quartile. Median follow-up durations ranged from 7.0 to 32.0 months. PFS was reported in seven studies [18, 20–25], and OS was reported in all eight studies [18–25]. Seven studies [18–23, 25] conducted multivariate analyses to assess the association between serum ferritin levels and survival outcomes, adjusting for variables, such as age, sex, extramedullary disease (EMD), and performance status to varying degrees. One study [24] used univariate analysis. The NOS scores for the included studies ranged from six to eight, indicating overall moderate to good study quality (Table 2).

**Table 2:** Study quality evaluation via the Newcastle-Ottawa Scale

**Study**	**Representativeness of the exposed cohort**	**Selection of the non-exposed cohort**	**Ascertainment of exposure**	**Outcome not present at baseline**	**Control for age and sex**	**Control for other confounding factors**	**Assessment of outcome**	**Enough long follow-up duration**	**Adequacy of follow-up of cohorts**	**Total**
Mohty, 2023	1	1	1	1	1	1	1	0 (<12 months)	1	8
Liu, 2023	0 (not consecutively or randomly included)	1	1	1	1	1	1	1	1	8
Dima, 2024	0 (not consecutively or randomly included)	1	1	1	1	1	1	1	1	8
Moreno, 2024	0 (not consecutively or randomly included)	1	1	1	0 (Univariate analysis only)	0 (Univariate analysis only)	1	1	1	6
Hashmi, 2024	0 (not consecutively or randomly included)	1	1	1	1	1	1	0 (<12 months)	1	7
Gagelmann, 2024	0 (not consecutively or randomly included)	1	1	1	1	1	1	0 (<12 months)	1	7
Davis, 2024	0 (not consecutively or randomly included)	1	1	1	1	1	1	0 (<12 months)	1	7
Sidana, 2025	0 (not consecutively or randomly included)	1	1	1	1	1	1	1	1	8

### Meta-analysis for the outcome of PFS

**Figure 2.**
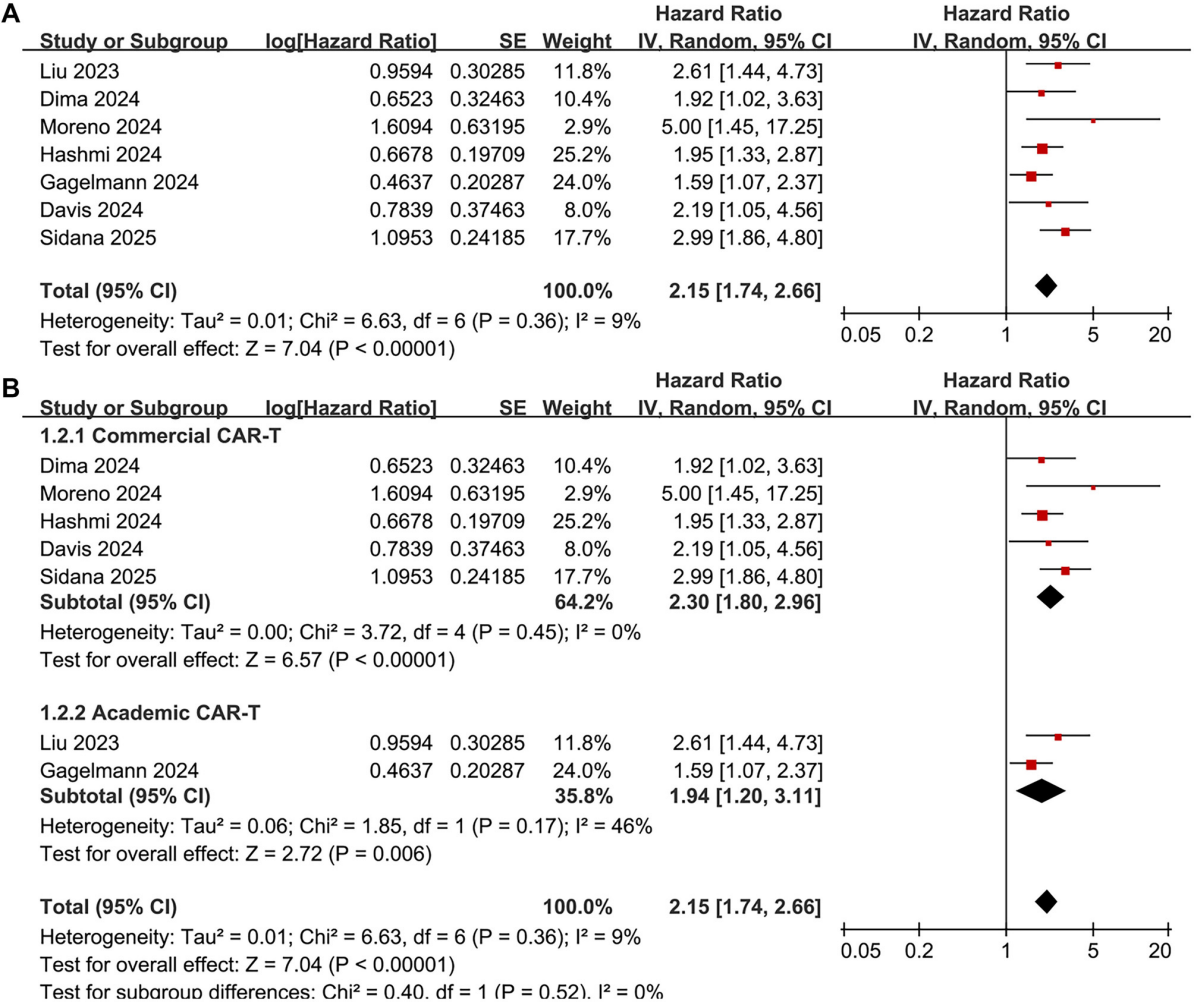
**Forest plots for the meta-analysis of the association between pre-infusion serum ferritin and PFS of R/R MM patients on CAR-T.** (A) Overall meta-analysis and (B) Subgroup analysis according to the source of CAR-T products. CAR-T: Chimeric antigen receptor-modified T-cell; CI: Confidence interval; R/R MM: Relapsed/refractory multiple myeloma; PFS: Progression-free survival.

**Figure 3.**
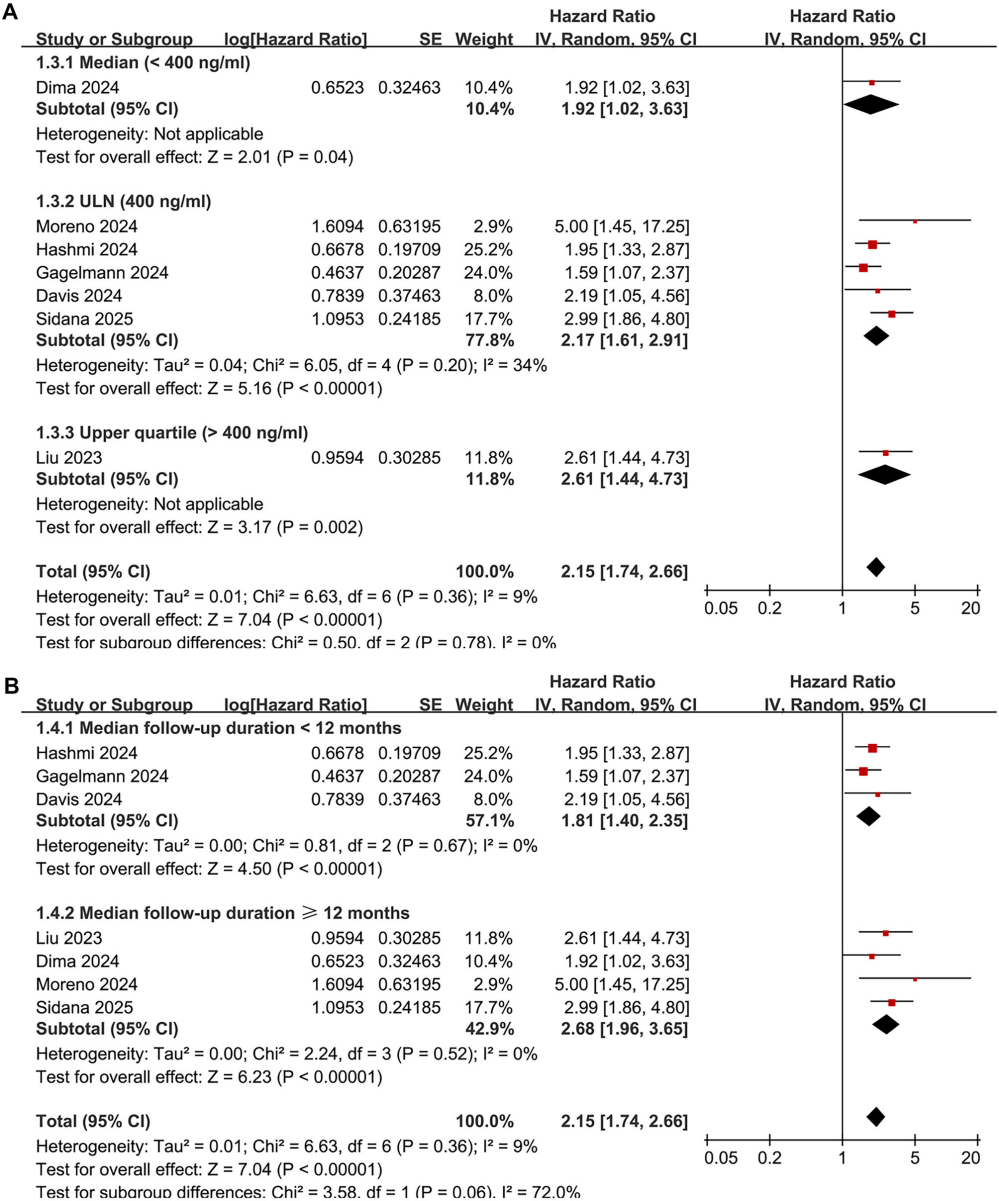
**Forest plots for the subgroup analyses of the association between pre-infusion serum ferritin and PFS of R/R MM patients on CAR-T.** (A) Subgroup analysis according to the cutoff of serum ferritin and (B) Subgroup analysis according to the follow-up durations. CAR-T: Chimeric antigen receptor-modified T-cell; CI: Confidence interval; R/R MM: Relapsed/refractory multiple myeloma; PFS: Progression-free survival.

Overall, seven studies [18, 20–25] reported an association between pre-infusion serum ferritin levels and PFS in patients with R/R MM treated with CAR-T therapy. Mild heterogeneity was observed among the studies (*I*^2^ ═ 9%). Pooled results using a random-effects model indicated that high pre-infusion serum ferritin levels were associated with poorer PFS (HR: 2.15, 95% CI: 1.74–2.66, *P* < 0.001; Figure 2A). Sensitivity analyses, conducted by excluding one dataset at a time, did not substantially alter the results (HR range: 1.98–2.35; all <0.05). Notably, excluding the only study based on univariate analysis [24] yielded similar findings (HR: 2.08, 95% CI: 1.70–2.55, *P* < 0.001; *I*^2^ ═ 0%). Subgroup analyses showed that the association between pre-infusion serum ferritin and poor PFS did not significantly differ between studies using commercial vs academic CAR-T products (*P* for subgroup difference ═ 0.52; Figure 2B), across studies using different ferritin cutoffs (*P* ═ 0.78; Figure 3A), or between studies with follow-up durations >12 months vs ≥12 months (*P* ═ 0.06; Figure 3B).

### Meta-analysis for the outcome of OS

**Figure 4.**
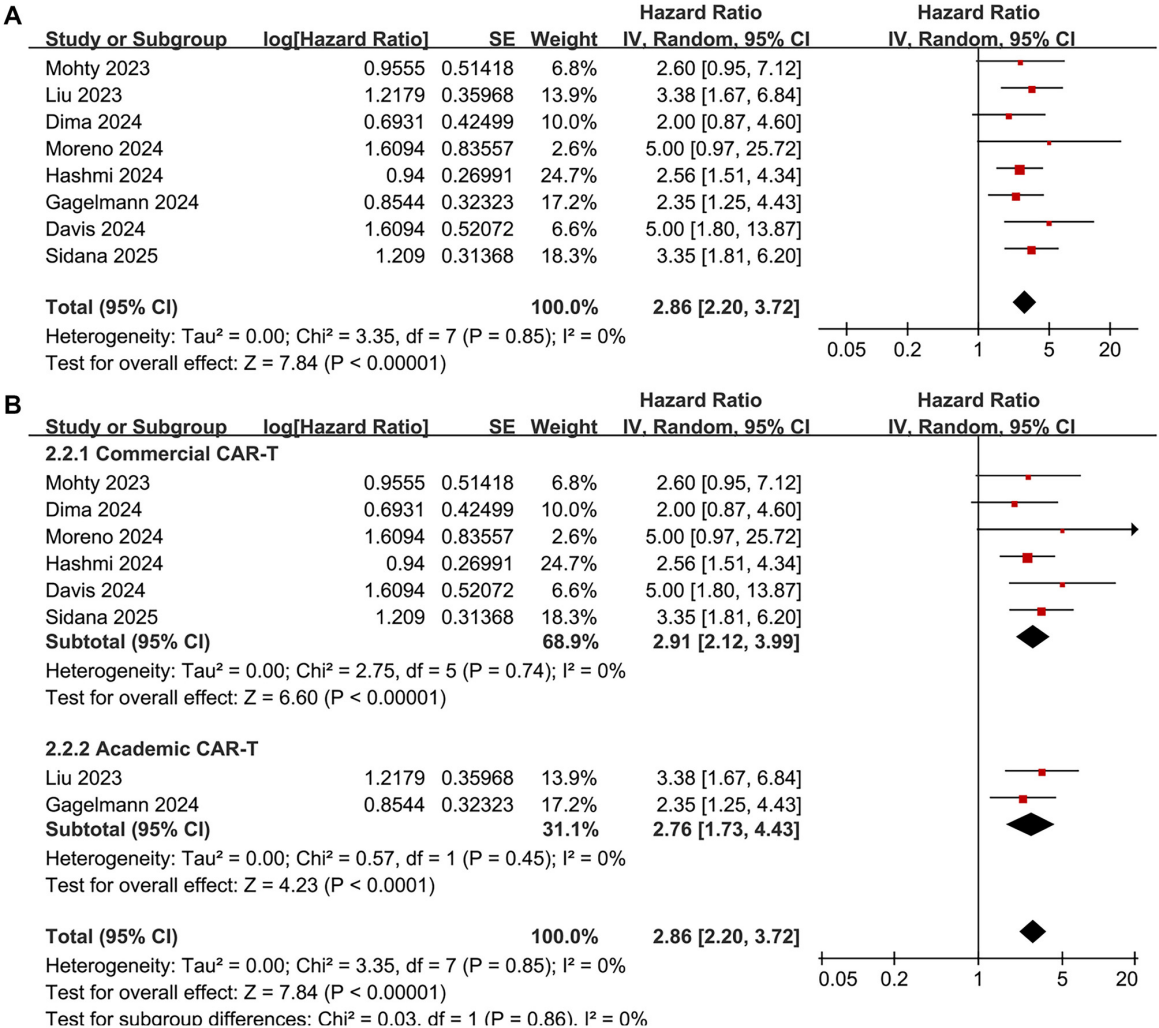
**Forest plots for the meta-analysis of the association between pre-infusion serum ferritin and OS of R/R MM patients on CAR-T.** (A) Overall meta-analysis and (B) Subgroup analysis according to the source of CAR-T products. CAR-T: Chimeric antigen receptor-modified T-cell; CI: Confidence interval; R/R MM: Relapsed/refractory multiple myeloma; OS: Overall survival.

**Figure 5.**
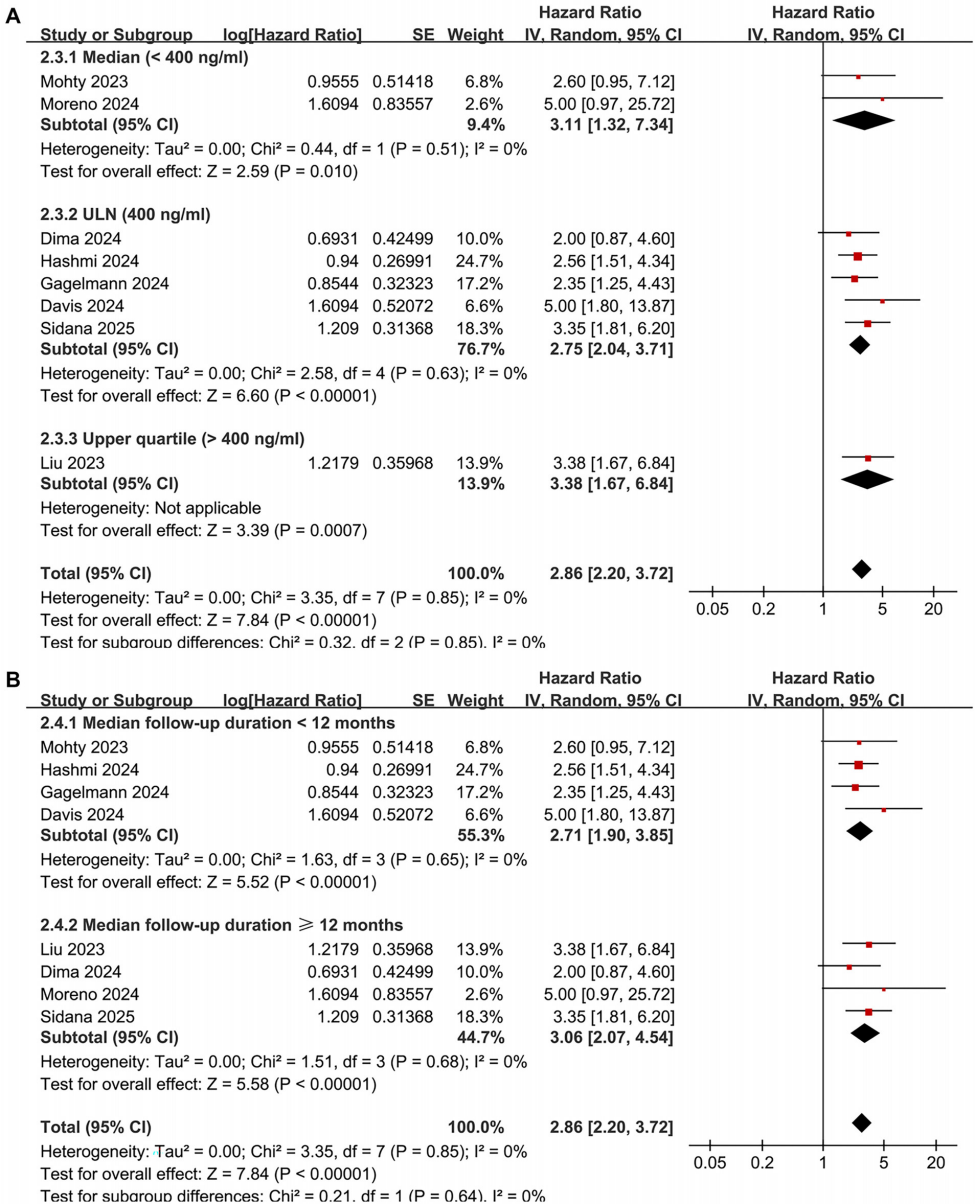
**Forest plots for the subgroup analyses of the association between pre-infusion serum ferritin and OS of R/R MM patients on CAR-T.** (A) Subgroup analysis according to the cutoff of serum ferritin and (B) Subgroup analysis according to the follow-up durations. CAR-T: Chimeric antigen receptor-modified T-cell; CI: Confidence interval; R/R MM: Relapsed/refractory multiple myeloma; OS: Overall survival.

The pooled results from eight studies [18–25] showed that a high serum ferritin level before CAR-T infusion was associated with poorer OS in patients with R/R MM during follow-up (HR: 2.86, 95% CI: 2.20–3.72, *P* < 0.001; Figure 4A), with no significant heterogeneity (*I*^2^ ═ 0%). Sensitivity analyses, conducted by omitting one study at a time, yielded similar results (HR range: 2.78–2.98, all *P* values <0.05). The findings remained consistent after excluding the only study [24] that used univariate analysis (HR: 2.82, 95% CI: 2.16–3.68, *P* < 0.001; *I*^2^ ═ 0%). Additional subgroup analyses based on CAR-T product source (*P* for subgroup difference ═ 0.86; Figure 4B), serum ferritin cutoffs (*P* ═ 0.85; Figure 5A), and median follow-up durations (*P* ═ 0.64; Figure 5B) also supported these consistent results.

### Publication bias

**Figure 6.**
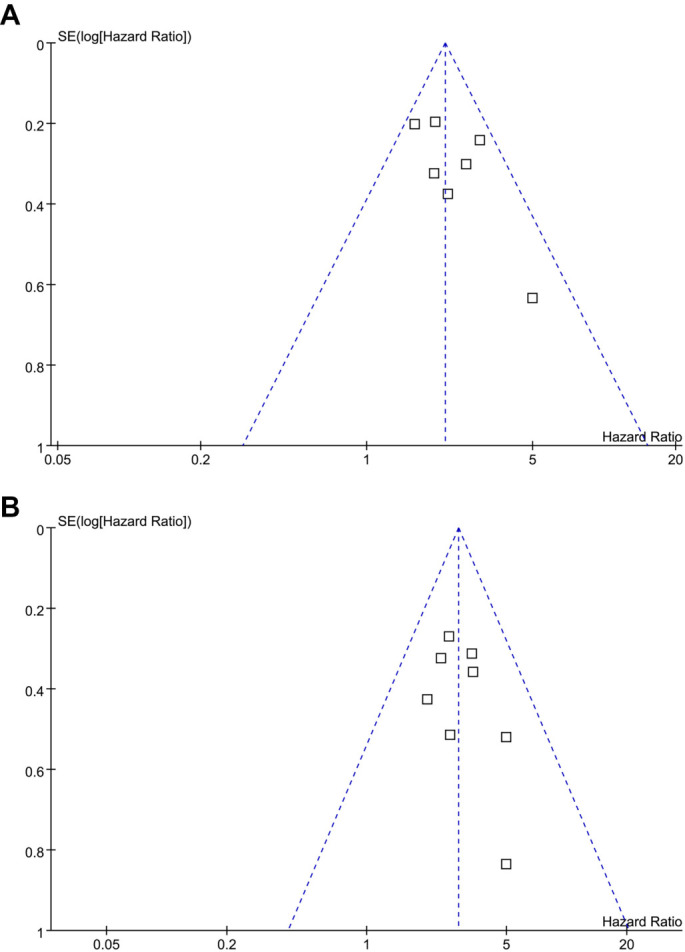
**Funnel plots for estimating the potential publication biases underlying the meta-analyses.** (A) Funnel plots for the meta-analysis of the association between pre-infusion serum ferritin and PFS of R/R MM patients on CAR-T and (B) Funnel plots for the meta-analysis of the association between pre-infusion serum ferritin and OS of R/R MM patients on CAR-T. CAR-T: Chimeric antigen receptor-modified T-cell; OS: Overall survival; R/R MM: Relapsed/refractory multiple myeloma; PFS: Progression-free survival.

The funnel plots for the meta-analyses examining the association between pre-infusion serum ferritin levels and survival outcomes in patients with R/R MM are shown in Figure 6A and 6B. Visual inspection of the plots reveals symmetry, suggesting a low risk of publication bias. This observation is supported by the results of Egger’s regression analyses, which also indicate a low risk of publication bias (*P* ═ 0.42 for PFS and *P* ═ 0.51 for OS).

## Discussion

The findings of this meta-analysis demonstrate a significant association between high pre-infusion serum ferritin levels and poor survival outcomes in patients with R/R MM treated with CAR-T therapy. Specifically, patients with elevated ferritin levels prior to CAR-T infusion exhibited a markedly increased risk of shorter PFS and OS. These associations remained robust across sensitivity analyses and subgroup analyses stratified by CAR-T product source, ferritin cutoff levels, and follow-up durations, with low or no heterogeneity observed. These results underscore the prognostic significance of serum ferritin in this clinical context, highlighting its potential as a biomarker for risk stratification. The mechanisms linking elevated ferritin levels to poor survival in CAR-T-treated R/R MM patients are complex and multifactorial. Pathophysiologically, ferritin reflects systemic inflammation and oxidative stress—hallmarks of advanced malignancy [32]. Elevated ferritin is associated with increased levels of pro-inflammatory cytokines, such as interleukin-6, which may exacerbate CAR-T-related toxicities, including CRS and ICANS [16]. These adverse events are critical determinants of survival, and their severity may be heightened in patients with high baseline ferritin levels [33]. Clinically, elevated ferritin may signal an immunosuppressive tumor microenvironment and impaired CAR-T cell functionality, potentially reducing therapeutic efficacy [34]. Moreover, high ferritin levels have been linked to increased risks of infections [35], cardiac events [36], cytopenia [37], and delayed platelet recovery [38] in R/R MM patients undergoing CAR-T therapy—all of which can negatively impact outcomes. Collectively, these findings support the role of elevated pre-infusion serum ferritin as a predictor of poor survival in patients with R/R MM receiving CAR-T therapy.

The results of the sensitivity analyses underscore the robustness of the findings, as excluding individual studies or focusing solely on multivariate analyses did not significantly alter the association between high ferritin levels and poor survival. Notably, a sensitivity analysis excluding the only study based on univariate analysis yielded consistent results, suggesting that the association between high ferritin and poor survival may be independent of factors, such as age, sex, presence of EMD, and performance status. Subgroup analyses further reinforced these findings, revealing no significant differences across CAR-T product sources (commercial vs academic), ferritin cutoffs, or follow-up durations. Collectively, these results indicate that the prognostic impact of ferritin is broadly applicable across diverse patient populations and treatment settings, supporting its potential clinical relevance.

This meta-analysis has several strengths. It presents an extensive and systematic evaluation of the most up-to-date evidence, incorporating a comprehensive literature search and rigorous adherence to PRISMA guidelines. By focusing on pre-infusion ferritin levels, the findings are made clinically actionable, as this biomarker can be readily assessed before the initiation of therapy. Additionally, the inclusion of multiple sensitivity and subgroup analyses enhances the robustness of the results, offering a more nuanced understanding of the factors that may influence the association between ferritin levels and survival outcomes.

Several limitations of this meta-analysis should be acknowledged. All included studies were retrospective in design, which may introduce recall and selection biases [39]. Consequently, the findings should be validated in prospective cohorts. Additionally, the systematic literature search was limited to PubMed, Embase, and Web of Science. While these databases are comprehensive, relevant studies indexed in other sources—such as the Cochrane Library or ClinicalTrials.gov—may have been missed. Future reviews should broaden the search scope to include additional databases and grey literature to ensure a more exhaustive identification of relevant studies. Although most included studies performed multivariate adjustments, unmeasured confounding factors may still have influenced the observed associations. Moreover, the use of study-level data in this meta-analysis precludes an assessment of individual patient characteristics—such as comorbidities, functional status, or genetic factors—which may affect the prognostic role of ferritin. For example, comorbid conditions like cardiovascular disease, chronic infections, or liver dysfunction could elevate ferritin levels independently of disease severity or systemic inflammation [40]. Functional status and frailty are also important considerations, as frailer patients may exhibit higher baseline inflammation and reduced tolerance to CAR-T therapy [20]. Furthermore, genetic and molecular features of multiple myeloma, including high-risk cytogenetics (e.g., del(17p), t(4;14)) or TP53 mutations, may influence both ferritin levels and clinical outcomes [41]. A key limitation is the focus on pre-infusion ferritin levels, without assessment of post-infusion changes. Monitoring ferritin kinetics over time—such as at days seven and 14 post-infusion—may offer additional prognostic insights, particularly in relation to treatment-related toxicities like CRS and ICANS. Prospective studies are needed to evaluate whether dynamic changes in ferritin levels can predict survival outcomes in CAR-T–treated patients. Another limitation is the lack of detailed information on the methods used to measure ferritin across studies. Variability in assay techniques may affect the comparability of results. Nonetheless, as all included studies were conducted in real-world clinical settings, ferritin was likely measured using standard laboratory protocols. Future research should report assay methodologies to improve consistency across studies. Importantly, the observational nature of the included studies precludes causal inference between elevated ferritin levels and poor survival outcomes.

Clinically, the results of this meta-analysis suggest that serum ferritin may serve as a useful prognostic biomarker in patients receiving CAR-T therapy for R/R MM. Elevated ferritin levels could help identify high-risk patients who may benefit from closer monitoring and more intensive supportive care during treatment. However, ferritin’s role remains limited to risk stratification, and it is premature to consider it a therapeutic target. Future research should focus on validating ferritin’s prognostic value in prospective studies, integrating it with other clinical and molecular markers, and evaluating its role in predicting CAR-T–related efficacy and toxicity. Additionally, it would be valuable to investigate whether patients with high pre-infusion ferritin levels could benefit from enhanced prophylactic strategies—such as anti-inflammatory agents—to reduce the risk of CRS and ICANS. While no direct evidence currently supports this approach, prospective studies employing ferritin-guided risk stratification may help optimize supportive care in CAR-T therapy.

## Conclusion

In conclusion, this meta-analysis provides up-to-date evidence that elevated pre-infusion serum ferritin levels are associated with poorer survival outcomes in patients with R/R MM undergoing CAR-T therapy. These findings highlight the potential of ferritin as a prognostic marker and offer valuable insights into its possible clinical utility. While further research is needed to address current limitations and refine its application, these results contribute to the growing body of knowledge aimed at improving patient outcomes in the era of CAR-T therapy.

## Supplemental data

**PubMed**

(“Ferritin”[Mesh] OR ferritin OR ferritins OR hyperferritinemia OR hyperferritinaemia) AND (“Multiple Myeloma”[Mesh] OR myeloma OR “multiple myeloma”) AND (“Chimeric Antigen Receptor T-Cell Therapy”[Mesh] OR “Chimeric Antigen Receptor T Cells”[Mesh] OR “CAR-T” OR “chimeric antigen receptor” OR “chimeric T cell receptor” OR “artificial T cell receptor” OR “axicabtagene ciloleucel” OR “axi-cel” OR “KTE-C19” OR “CTL-019” OR “yescarta” OR “lisocabtagene maraleucel” OR “liso-cel” OR “breyanzi” OR “brexucabtagene autoleucel” OR “brexu-cel” OR “tecartus” OR “tisagenlecleucel” OR “tisa-cel” OR “kymriah” OR “idecabtagene vicleucel” OR “cilta-cel” OR “Ciltacabtagene autoleucel”)

**Embase**

(“ferritin”/exp OR ferritin OR ferritins OR hyperferritinemia OR hyperferritinaemia) AND (“multiple myeloma”/exp OR myeloma OR “multiple myeloma”) AND (“chimeric antigen receptor”/exp OR “CAR T cell”/exp OR “chimeric antigen receptor t cells” OR “chimeric T cell receptor” OR “artificial T cell receptor” OR “axicabtagene ciloleucel” OR “axi-cel” OR “KTE-C19” OR “CTL-019” OR “yescarta” OR “lisocabtagene maraleucel” OR “liso-cel” OR “breyanzi” OR “brexucabtagene autoleucel” OR “brexu-cel” OR “tecartus” OR “tisagenlecleucel” OR “tisa-cel” OR “kymriah” OR “idecabtagene vicleucel” OR “cilta-cel” OR “Ciltacabtagene autoleucel”)

**Web of Science**

TS=(“ferritin” OR “ferritins” OR “hyperferritinemia” OR “hyperferritinaemia”) AND TS ═ (“myeloma” OR “multiple myeloma”) AND TS ═ (“Chimeric Antigen Receptor” OR “CAR-T” OR “chimeric antigen receptor T cells” OR “chimeric T cell receptor” OR “artificial T cell receptor” OR “axicabtagene ciloleucel”” OR “axi-cel” OR “KTE-C19” OR “CTL-019” OR “yescarta” OR “lisocabtagene maraleucel” OR “liso-cel” OR “breyanzi” OR “brexucabtagene autoleucel” OR “brexu-cel” OR ””tecartus” OR “tisagenlecleucel”” OR “tisa-cel” OR ”kymriah” OR “idecabtagene vicleucel” OR “cilta-cel” OR “Ciltacabtagene autoleucel”)

## Data Availability

The data that support the findings of this study are available from the corresponding author upon reasonable request.
